# Kasabach–Merritt phenomenon with concurrent appearance of antiphospholipid antibodies in a patient with giant liver haemangioma

**DOI:** 10.1093/rap/rky047

**Published:** 2018-10-24

**Authors:** Hiroyuki Nakamura, Yuichiro Fujieda, Masaru Kato, Tatsuya Atsumi

**Affiliations:** Department of Rheumatology, Endocrinology and Nephrology, Faculty of Medicine and Graduate School of Medicine, Hokkaido University, Sapporo, Japan

**Key message**

- Appearance of antiphospholipid antibodies may play a role in development of Kasabach–Merritt phenomenon.

Sir, aPL refers to autoantibodies against plasma proteins binding anionic phospholipids, such as cardiolipin (CL) and phosphatidylserine. β2-Glycoprotein I (β_2_GPI), a circulating protein mainly produced by hepatocytes, is one of the major antigens of aPL. The persistent presence of aPL is associated with a risk of recurrent thrombotic events and obstetric complications, known as APS. However, it has been unclear how β_2_GPI plays a role as an autoantigen. Here, we describe an interesting case of giant liver haemangioma, in which thrombocytopenia and consumptive coagulopathy, namely Kasabach–Merritt phenomenon, occurred in parallel to transient appearance of aPL. We discuss a possible mechanism of aPL production and the pathophysiology of Kasabach–Merritt phenomenon through this clinical course.

A 48-year-old woman visited our clinic with fever, cough and runny nose lasting for the past 1 week, suggesting viral infection in the upper respiratory tract. She had liver haemangioma (Fig. 1B) and a 15-year history of recurrent thrombocytopenia. A thrombocytopenia (2.2 × 10^4^ /μl) was associated with this upper respiratory infection. She had positive ANA (1/640), a high titer of anti-CL/β_2_GPI antibodies (27.8 U/ml, reference, <3.5 U/ml) and positivity of lupus anticoagulant. Thus, she had high total aPL score, a predictive marker of thrombosis in autoimmune diseases, as previously reported [1]. However, she did not fulfil the classification criteria for SLE or APS because she did not have anti-DNA antibodies, hypocomplementaemia, any other organ dysfunction, thrombosis or obstetric complications. In the past, her thrombocytopenia was successfully treated with 60 mg/day of prednisolone, accompanied by a reduction in the aPL score, and had been maintained by 5 mg/day of prednisolone.

**Fig. 1:**
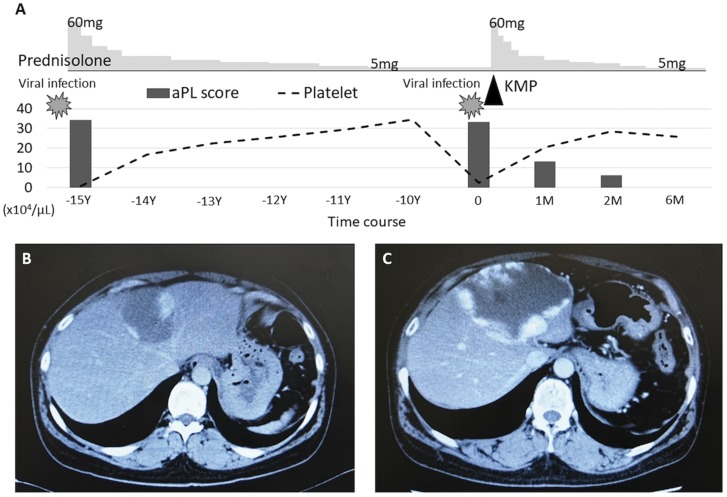
Clinical course and laboratory findings (**A**) The aPL score was calculated according to type of positive aPL test and their titers. Liver contrast-enhanced CT images in the first (**B**) or second (**C**) episode of thrombocytopenia. Peripheral enhancement of the hypoattenuating liver tumours indicates haemangioma. KMP: Kasabach–Merritt phenomenon.

On this visit, laboratory examinations showed a relapse of thrombocytopenia and the following coagulation disorders: fibrin degradation products, 186.0 μg/ml (reference, <10.0 μg/ml); D-dimer, 75.2 μg/ml (reference, <1.0 μg/ml); fibrinogen, 132 mg/dl (reference, 150–400 mg/dl); prothrombin time, 13 s (reference, 10–12 s); and aPTT, 74 s (reference, 26–42 s). Anti-CL/β_2_GPI complex antibodies and lupus anticoagulant reappeared in her plasma; her total aPL score increased. Contrast-enhanced CT revealed a markedly enlarged giant liver haemangioma (Fig. 1C). High-dose prednisolone (60 mg/day) improved her thrombocytopenia and coagulation disorder. The aPL score again decreased (Fig. 1A).

We previously reported via *in vitro* studies that β_2_GPI was presented by activated vascular endothelial cells as β_2_GPI/HLA class II complexes [2]. Endothelial cells have the potential ability to present antigen through HLA class II expression induced by inflammatory cytokines, such as IFN-γ and TNF-α. Therefore, cytokines produced in response to infection might induce the formation of β_2_GPI/HLA class II complexes on endothelial cells and might trigger aPL production. This patient had a giant liver haemangioma, with hyperplasia of hepatic endothelial cells. The presence of excessive endothelial cells as antigen presenting cells might promote the production of aPL, although only one case of liver haemangioma with aPL has been reported [3]. In addition, it has been shown that a difference in HLA class II alleles affects T cell development, conferring susceptibility to autoimmune diseases [2]. The patient had HLA-DRB1 15:01/15:02 alleles, which is reported to be associated with autoimmune diseases [4]. The type of her HLA-DRB1 alleles might lead to conditions in which it was easy to produce autoantibodies when the endothelial cells were activated by cytokines.

We also showed that aPL have cytotoxicity against endothelial cells expressing the β_2_GPI/HLA class II complexes [2]. aPL induce strong complement-mediated injury of binding endothelial cells and promote tissue factor production via the p38 mitogen-activated protein kinase pathway and nuclear factor-κB [5]. Injured endothelial cells with tissue factors aggregate platelets and activate the coagulation cascade. Consumptive thrombocytopenia is often found in aPL-positive patients and is proposed as a predictive factor of thrombosis [6]. Kasabach–Merritt phenomenon (thrombocytopenia and consumptive coagulopathy) typically occurs in children with kaposiform haemangioendotheliomas or tufted angiomas, and rarely develops in adults with giant liver haemangiomas. The pathophysiology is reckoned to be platelet trapping and consumption within the abnormal vasculature [7]. Aggregated platelets subsequently activate the coagulation cascade, leading to consumptive coagulopathy. Endothelial cells derived from haemangioma have a more procoagulant and angiogenic phenotype compared with normal endothelial cells [8]. In this case, thrombocytopenia and coagulation disorder were not found in a steady state, but occurred only after the episodes of viral infection. Kasabach–Merritt phenomenon is sometimes induced by infection, as in this case, because endothelial cells activated and injured by inflammatory cytokines promote platelet trapping and aggregation [7]. Additionally, in this case, appearance of aPL could play an important role in the development of Kasabach–Merritt phenomenon through aPL-mediated endothelial cell injury.

We think that this clinical course would support our *in vitro* hypothesis about aPL production and pathogenicity. Glucocorticoids are the first choice for treating Kasabach–Merritt phenomenon to suppress production of cytokines and inhibit excessive activation of platelets and endothelial cells [7]. In this patient, resection of the giant liver haemangioma could be considered, because the haemangioma could be the origin of aPL production and the Kasabach–Merritt phenomenon.

*Funding*: No specific funding was received from any funding bodies in the public, commercial or not-for-profit sectors to carry out the work described in this manuscript.

*Disclosure statement*: The authors have declared no conflicts of interest.

## References

[1] Otomo K, Atsumi T, Amengual O et al Efficacy of the antiphospholipid score for the diagnosis of antiphospholipid syndrome and its predictive value for thrombotic events. Arthritis Rheum 2012;64:504–12. 21953404 10.1002/art.33340

[2] Tanimura K, Jin H, Suenaga T et al β2-Glycoprotein I/HLA class II complexes are novel autoantigens in antiphospholipid syndrome. Blood 2015;125:2835–44. 25733579 10.1182/blood-2014-08-593624 PMC4424631

[3] Maeshima E, Minami Y, Sato M et al A case of systemic lupus erythematosus with giant hepatic cavernous hemangioma. Lupus 2004;13:546–8. 15352428 10.1191/0961203303lu1040oa

[4] Kuwana M, Kaburaki J, Arnett FC et al Influence of ethnic background on clinical and serologic features in patients with systemic sclerosis and anti-DNA topoisomerase I antibody. Arthritis Rheum 1999;42:465–74. 10088769 10.1002/1529-0131(199904)42:3<465::AID-ANR11>3.0.CO;2-Y

[5] Oku K, Amengual O, Zigon P et al Essential role of the p38 mitogen-activated protein kinase pathway in tissue factor gene expression mediated by the phosphatidylserine-dependent antiprothrombin antibody. Rheumatology 2013;52:1775–84. 23878313 10.1093/rheumatology/ket234

[6] Hisada R, Kato M, Sugawara E et al Thrombotic risk stratification by platelet count in patients with antiphospholipid antibodies: a longitudinal study. J Thromb Haemost 2017;15:1782–7. 28662299 10.1111/jth.13763

[7] O’Rafferty C, O’Regan GM, Irvine AD, Smith OP. Recent advances in the pathobiology and management of Kasabach–Merritt phenomenon. Br J Haematol 2015;171:38–51. 26123689 10.1111/bjh.13557

[8] Zhang WJ, Ye LY, Wu LQ et al Morphologic, phenotypic and functional characteristics of endothelial cells derived from human hepatic cavernous hemangioma. J Vasc Res 2006;43:522–32. 17008795 10.1159/000095965

